# Arrowtail RNA for Ligand Display on Ginger Exosome-like Nanovesicles to Systemic Deliver siRNA for Cancer Suppression

**DOI:** 10.1038/s41598-018-32953-7

**Published:** 2018-10-02

**Authors:** Zhefeng Li, Hongzhi Wang, Hongran Yin, Chad Bennett, Huang-ge Zhang, Peixuan Guo

**Affiliations:** 1 Center for RNA Nanobiotechnology and Nanomedicine, College of Pharmacy, Division of Pharmaceutics and Pharmaceutical Chemistry, College of Medicine, Dorothy M. Davis Heart and Lung Research Institute, NCI Comprehensive Cancer Center, The Ohio State University, Columbus, OH 43210 USA; 2 Medicinal Chemistry Shared Resource, Comprehensive Cancer Center, The Ohio State University, Columbus, OH 43210 USA; 3 James Brown Cancer Center, Department of Microbiology & Immunology, University of Louisville, Louisville, KY USA

## Abstract

Exosomes have shown increasing potential as delivery vesicles for therapy, but challenges like cost/yield, drug payload, and targeting specificity still exist. Plant derived exosome-like nanoparticles have been reported as a promising substitution and exhibit biocompatibility through oral, intranasal administration; however, systemic delivery of siRNA by exosome-like nanoparticles directly isolated from plants has not been reported. Recently, we reported the control of RNA orientation to decorate human derived exosome with cell targeting ligands for specific delivery of siRNA to tumors. Here, we expand to the application of arrowtail RNA nanoparticles for displaying ligands on ginger derived exosome-like nanovesicles (GDENs) for siRNA delivery and tumor inhibition through IV administration. Cushion ultracentrifugation coupled with equilibrium density gradient ultracentrifugation were used for purifying GDENs that displayed size, density, and morphology similar to human derived exosomes. Folic acid (FA), as a ligand, was displayed on the surface of GDENs for targeted delivery of survivin siRNA to KB cancer models. *In vitro* gene knockdown efficacy by FA-3WJ/GDENs/siRNA complex was comparable to transfection. We observed inhibition of tumor growth on a xenograft model by intravenous administration, which reveals the potential of GDENs as an economic delivery system for siRNA.

## Introduction

Extracellular Vesicles (EVs), especially exosomes, have been reported as a type of potential delivery vehicle for therapy^1–5^. Its advantages include the fact that they are natural carriers of proteins and RNAs^6–8^. They can carry high payloads while remaining a favorable size (40~150 nm) and are well-tolerated *in vivo*^5,9^. Human cell culture medium is one of the major EV sources, however, methods of production and scaling up remain challenging. Several strategies, including the use of bioreactors, have been developed to scale up the production^10^, but economically, the cost/yield ratio is still not favorable for clinical application.

An emerging solution to the aforementioned problem is to harvest EVs or exosome-like vesicles from substituted sources including human urine^11^, bovine milk^12^ and especially plants^13,14^. Exosome-like nanometer sized particles holding similar properties as mammalian EVs have been reported in grapefruit^15^, grapes^16^, ginger^17,18^, sunflowers^19^, carrots, etc^20,21^. Compared to mammalian cell culture medium, plants are an advantageous source to scale up overall EV yield. Several studies have demonstrated that exosome-like vesicles from edible plants can be used for therapeutic or delivery purposes by oral^15,18^ or intranasal administration^16^. But intravenous injection studies of plant derived exosome-like vesicles are rarely reported. One of the major concerns is the biocompatibility regarding the particle size and impurity. One reported solution is to reassemble nanoparticles after extracting the lipid components from grapefruit, enabling the encapsulation and delivery of chemical drugs or miRNAs via intravenous route^22–24^.

When using biosynthetic materials for cancer therapy, tumor targeting specificity is an important consideration. In comparison to EVs derived from mammalian cells, plant exosome-like vesicles do not have ligands for cell targeting. Previous achievement in targeting of plant exosome-like vesicles is through its natural biodistribution properties to reach liver and intestines^15,17,18^. Endogenous engineering strategies, such as fusing of ligand protein^1^ during EV biogenesis have been reported. But this technology is difficult to be applied to plant exosome-like vesicles. In combating the targeting specificity issue, RNA nanotechnology has been proven to be a versatile and biocompatible platform for specific targeted therapeutic use^25–29^. The ultra-thermostable pRNA-3WJ core, identified in bacteriophage phi29 DNA packaging motor, has been used in many applications, including target specific delivery, controlled drug release, and image guided diagnostics, etc^26,28,30–34^. Here we adapted the post-biogenesis method of RNA nanotechnology we recently reported to manipulate the angle and orientation of the RNA architecture for displaying ligands onto the EVs surface to enhance targeting specificity^5^. Using ginger derived exosome-like nanovesicles (GDENs), we further confirm that exosome-like vesicles can be engineered via ligand-displaying arrowtail RNA nanoparticles to deliver siRNA for tumor suppression intravenously.

## Results

### Purification of Ginger Derived Exosome-like Nanovesicles (GDENs) according to density and size

**Figure 1:**
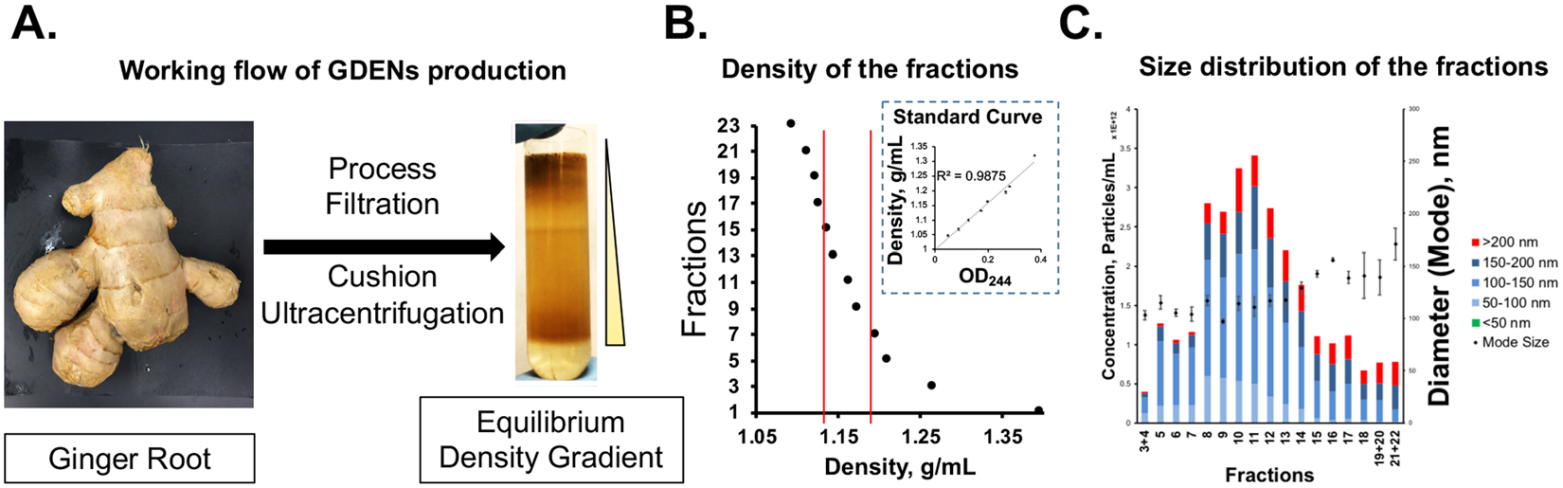
Purification and characterization of GDENs. (**A**) Working flow of purify GDENs from ginger root. (**B**) Density assessment of each fraction collected from equilibrium density gradient measured by OD244 and converted by standard curve (Fig. S1). Density of fraction 8~15 are located within the range of 1.13~1.19 g/mL (indicated by two red lines). (**C**) Size distribution and particle concentration of each fraction measured by NTA and plot with the mode size, original NTA result shown in Fig. S2. DLS characterization of final GDENs product are available in Fig. S3.

Methods of membrane filtration, differential ultracentrifugation, and equilibrium density gradient ultracentrifugation were used as a workflow to isolate GDENs (Fig. 1A). After ginger juice was blended, larger solid residues were removed by rough filtration, followed by the removal of cells and cell debris through centrifugation at 10,000 g twice. Crude GDENs were concentrated by repeated ultracentrifugation with the addition of a thin Optiprep^TM^ cushion at the bottom of the centrifuge tube, eliminating disruption and aggregation^5^. Since exosomes are complex lipid vesicles containing both protein and RNA, they have higher density than other lipid vesicles. Therefore, density gradient was chosen to separate exosome from free lipid, protein, RNA and other components. Condensed GDENs were further purified by equilibrium density ultracentrifugation and fractionated from the bottom of the tube^35^. The density among fractions indicates the gradient formed continuously and linearly (Figs 1B and S1). Fractions 8 ~ 15 were selected based on their density within the density ranges of HEK293T cell derived exosomes between 1.13–1.19 g/mL^36^. Particles concentration of each fraction by Nanoparticle Tracking Analysis (NTA) showed that most of the nanovesicles were distributed among fractions 8–14 (Figs 1C and S2). These fractions were then combined into one batch and washed by cushion ultracentrifugation in 1 × PBS again and resuspended in 1 × PBS for further application.

### Improvement of GDENs yield and reservation of authentic structure by applying isosmotic buoyant density and isosmotic cushion ultracentrifugation

**Figure 2:**
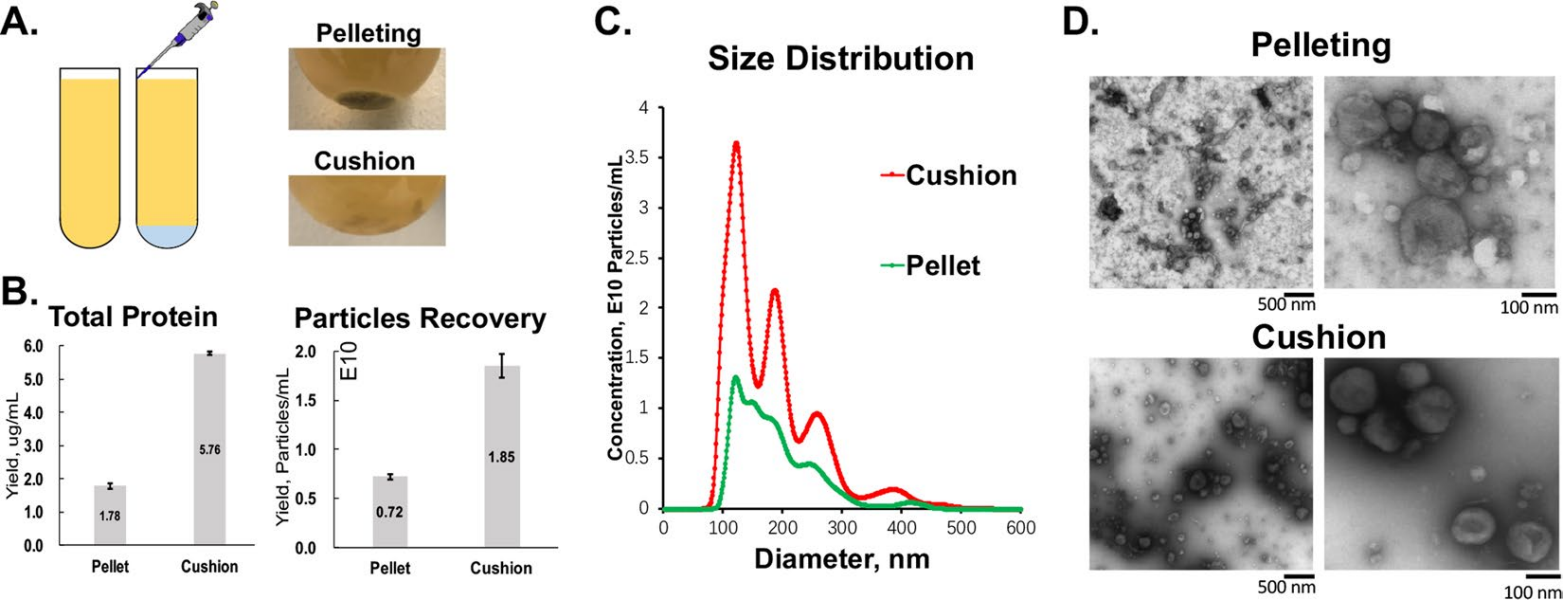
Characterization and comparison between ultracentrifugation with and without cushion for GDENs purification. (**A**) Schematic of cushion load to ultracentrifuge tube by slowly pipetting from side wall to the bottom and photo shows GDENs concentrated at the interception of cushion layer compare to the pellet pack firmly at the bottom. (**B**) Comparison of yield by total protein and particles standardized by volume of ginger juice isolated from. (**C**) Size distribution and particle concentration measured by NTA of GDENs purified from equal amount of ginger juice with and without cushion. (**D**) Negative staining TEM imaging showing the morphology of GDENs purified by pelleting and cushion method.

Ultracentrifugation is a common method for EVs purification. However, repeated pelleting of exosomes under high centrifuge force may damage the EVs integrity or cause aggregation^37^. By placing a thin layer of high-density iso-osmotic material at the bottom, EVs will never spinout as a pellet at the bottom of the centrifuge tube^10,35^. In this study, we also took advantage of Optiprep cushion during ultracentrifugation (Fig. 2A). It revealed that the cushion method increased not only yield but also the quality of GDENs. As shown in Fig. 2B, NTA and bicinchoninic acid (BCA) assay both indicated that GDENs harvested by cushion centrifugation have more than 2-fold higher yield than pelleting. No significant difference in size was observed between these two methods (cushion: 123.5 nm vs. pellet: 124.5 nm) but the distribution of GDENs from cushion seems to be less heterogeneous (Fig. 2C). We also performed negative staining TEM imaging to characterize the morphology of GDENs we purified (Fig. 2D). It revealed that GDENs purified with cushion had a cleaner background, less aggregation, and reserved a better spherical shape compared to conventional pelleting without a cushion. Therefore, the addition of the cushion during GDENs purification can eliminate the structural disruption and aggregation during ultracentrifugation.

### Loading of Exogenous Therapeutic RNA into GDENs

**Figure 3:**
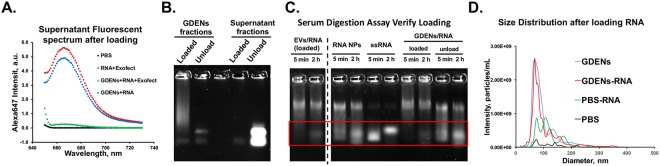
Loading RNA into GDENs. (**A**) Alexa_647_ spectrum of the supernatant fraction after loading for loading efficiency estimation. (**B**) Equal amount of GDENs fraction and supernatant fraction were loaded in a 2% Synergel for electrophoresis and visualize by Alexa_647_ channel. (**C**) 2% Synergel image showed GDENs loaded with 3WJ-Alexa_647_ undergo 67% serum digestion compare to control groups. (All lanes displayed are in a same gel, full length gel in Supplementary Fig. S4). (**D**) NTA measurement of size distribution and particle quantification of RNA loaded GDENs compare to control groups.

It is important to evaluate whether therapeutic cargo can be loaded into GDENs. Baculoviral inhibitor of apoptosis repeat-containing 5 (*BIRC5*), also known as survivin, is proven to be a promising target for cancer therapy; as knocking down survivin by RNAi can decrease tumorigenicity and inhibit metastases^5,38^. To improve the stability of siRNA *in vivo*, pyrimidines were 2′F-modified on the passenger strand to provide RNase resistance, while the guide strand was kept unmodified. For tracking siRNA loading efficiency in EVs, the survivin siRNA was fused to an Alexa_647_-labelled 3WJ core then was loaded into exosomes following procedures previously reported^5^. In short, 3WJ-Alexa_647_ mixed with equal volume of 1 × PBS and treated with Exofect was used as background to evaluate loading efficacy. About 80% decrease of the fluorescent signal in the supernatant was seen after loading compared to “GDENs + RNA” control groups (Fig. 3A). This indicated that most of the RNA molecules co-precipitated with the GDENs as a result of the loading procedures rather than non-specific binding. The loading efficiency was 80%. To further distinguish between the RNA loading and the aggregation with GDENs, equal amount of loaded and unloaded samples were run on electrophoretic gel (Fig. 3B). Due to pore size restriction, RNA loaded in GDENs was barely able to run into the gel, while unloaded RNA or co-precipitated RNA with GDENs by non-specific interaction can run into the gel under electrophoretic forces. To further confirm RNA loading, serum digestion assay were performed to verify that GDENs should protect RNA cargo against RNase digestion. Figure 3C, lanes 3–6 represented the free RNA digestion pattern under 2 hours treatment with 67% FBS while lanes 1&2 showed that the loaded RNA was protected by EVs. Loading of RNA into GDENs resulted in similar protection effect (lane 7&8) while RNA degradation was observed by simply mixing RNA with GDENs (lane 9&10). This data confirms the loading efficacy and distinguishes the loading of RNAs into GDENs from the non-specific interactions. In addition, NTA results indicated no significant change in particle sizes between GDENs and GDENs after loading, while treating the RNA with “Loading reagent only” showed completely different patterns on size distribution and particle concentration (Fig. 3D).

### Displaying of ligands on GDENs using Arrow-tail RNA nanoparticles for specific cancer-targeting

**Figure 4:**
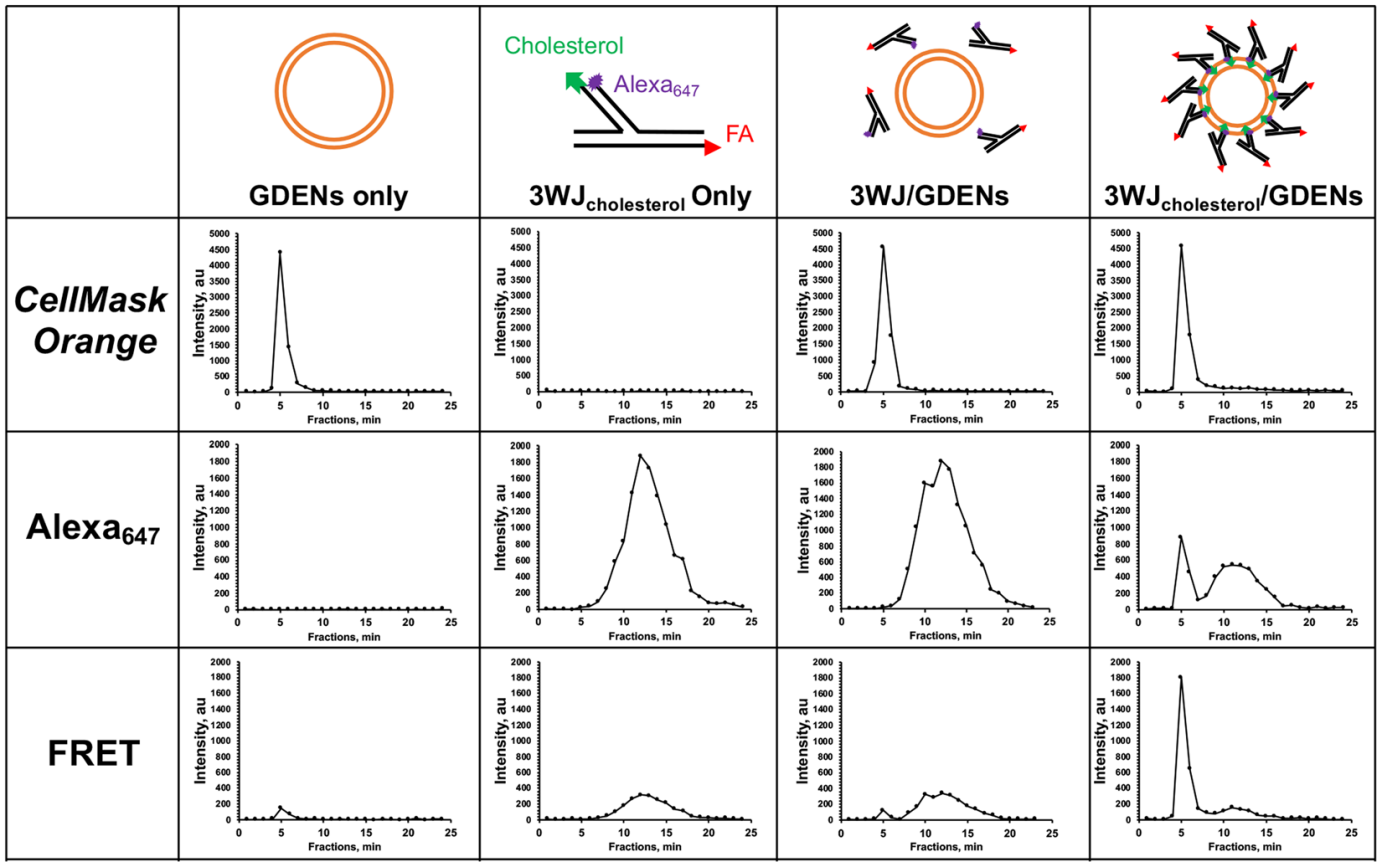
Histogram plotted by fluorescent intensity readout from individual fractions collected from Sephadex G-200 SEC after introduce sample by Cellmask Orange, Alexa_647_ and Cellmask Orange-Alexa_647_ FRET excitation/emission setting.

We recently reported that cholesterol-conjugated pRNA-3WJ can be used to decorate EVs with cancer specific ligands to target cancer cells and solid tumors in xenograft models^5^. To testify whether we can adopt the same strategy for engineering GDENs, we first applied Förster Resonance Energy Transfer (FRET) system to verify the interactions between RNA and GDENs. According to the mechanism of FRET, when applying a laser to excite the donor fluorophore, a receptor fluorophore then receives energy transfer and emits fluoresce. Due to sensitivity, FRET can only occur when the two fluorophores are within 10 nm distance^39–42^. GDENs were labeled by *CellMask Orange*, a uniform membrane labeling marker, with similar fluorescence emission spectrum as Cy3, to serve as a FRET donor. The arrowtail pRNA-3WJ was designed as a FRET accepter by end-labeling with a fluorescent dye Alexa_647_ on the 5′ end of 3WJ_b_ strand, which is adjacent to the cholesterol responsible for interactions with GDENs’ membrane (Fig. 4). The 2′F-modified Alexa_647_ labeled arrowtail pRNA-3WJ was incubated with *CellMask Orange* labeled GDENs forming complexes. The 3WJ/GDENs complex was fractionated by *Sephadex* G-200 gravity size exclusion column and compared to control groups. Fluorescent signals of *CellMask Orange*, Alexa_647_, and *CellMask Orange*-Alexa_647_ FRET were observed and plotted (Fig. 4**)**. The ~100 nm GDENs (5 min) were clearly separated from arrowtail pRNA-3WJ (11–13 min), which has been reported to be around 5 nm in size^43^. Only pRNA-3WJ-cholesterol/GDENs group showed a peak in 5 min fraction, indicating the RNA colocalized with GDENs as it passed through the column. A significant FRET peak compared to control groups confirms the pRNA-3WJ-cholesterol interacted with the GDENs membrane rather than non-specific effect.

**Figure 5:**
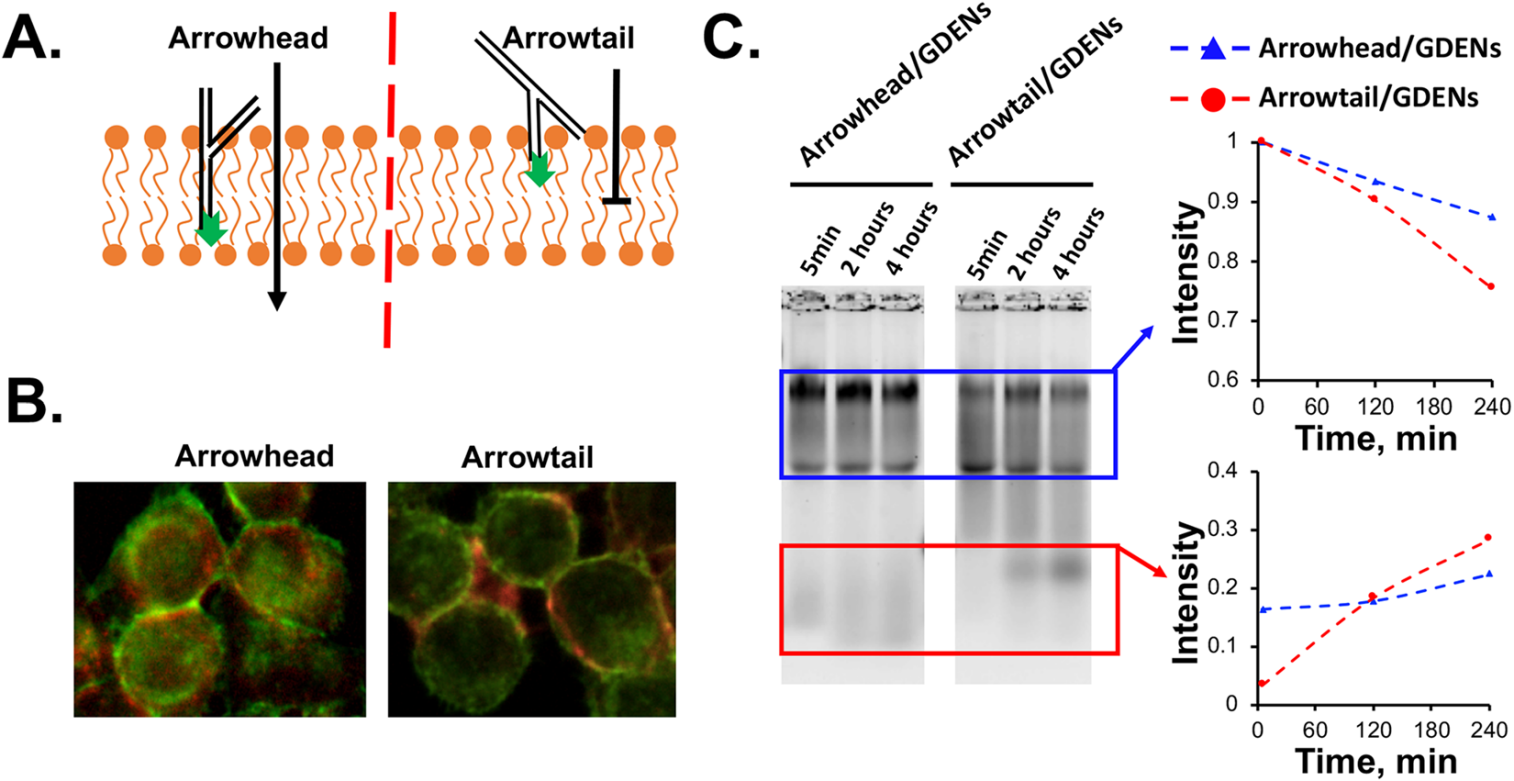
The interaction of arrowtail and arrowhead-displaying GDENs with cells. (**A**) Concept of ligand displaying by arrowtail and partially loading by arrowhead cause by different orientation. (**B**) Alexa_647_ labeled arrowtail and arrowhead RNA nanoparticle (red) incubate with KB cell (green) and image by fluorescent microscopy as model to show surface displaying and loading. (**C**) Serum digestion assay indicate partially loaded arrowhead RNA nanoparticle exhibit more resistant than surface displaying arrowtail RNA nanoparticle. Band intensity were quantified by ImageJ and standardized by 5 min digestion sample.

As reported in our recent publication, orientation of RNA arrow-shape RNA nanoparticles has different interaction patterns with membrane of EVs^5^. The head and tail configuration of the arrow-shaped pRNA-3WJ nanostructure will result in different physical hindrances when interacting with the lipid bilayer that had partial loading or surface display pattern (Figs 5A and S5). We used KB cells as a model to prove this concept since ~100 nm size GDENs was challenging to image by microscopy. Alexa_647_ labeled arrowhead RNA (red) distributed and internalized in cell (green) while arrowtail RNA displayed on the surface of cell membrane (Fig. 5B). In addition, serum digestion was used to verify the entry or surface display of the arrow-shape pRNA-3WJ nanoparticles for GDENs following our previous procedures^5^. Aarrowhead/GDENs was degraded with lower efficiency comparing to arrowtail/GDENs, indicating that more arrowtail RNA than arrowhead RNA were exposed to RNase in serum (Fig. 5C).

**Figure 6:**
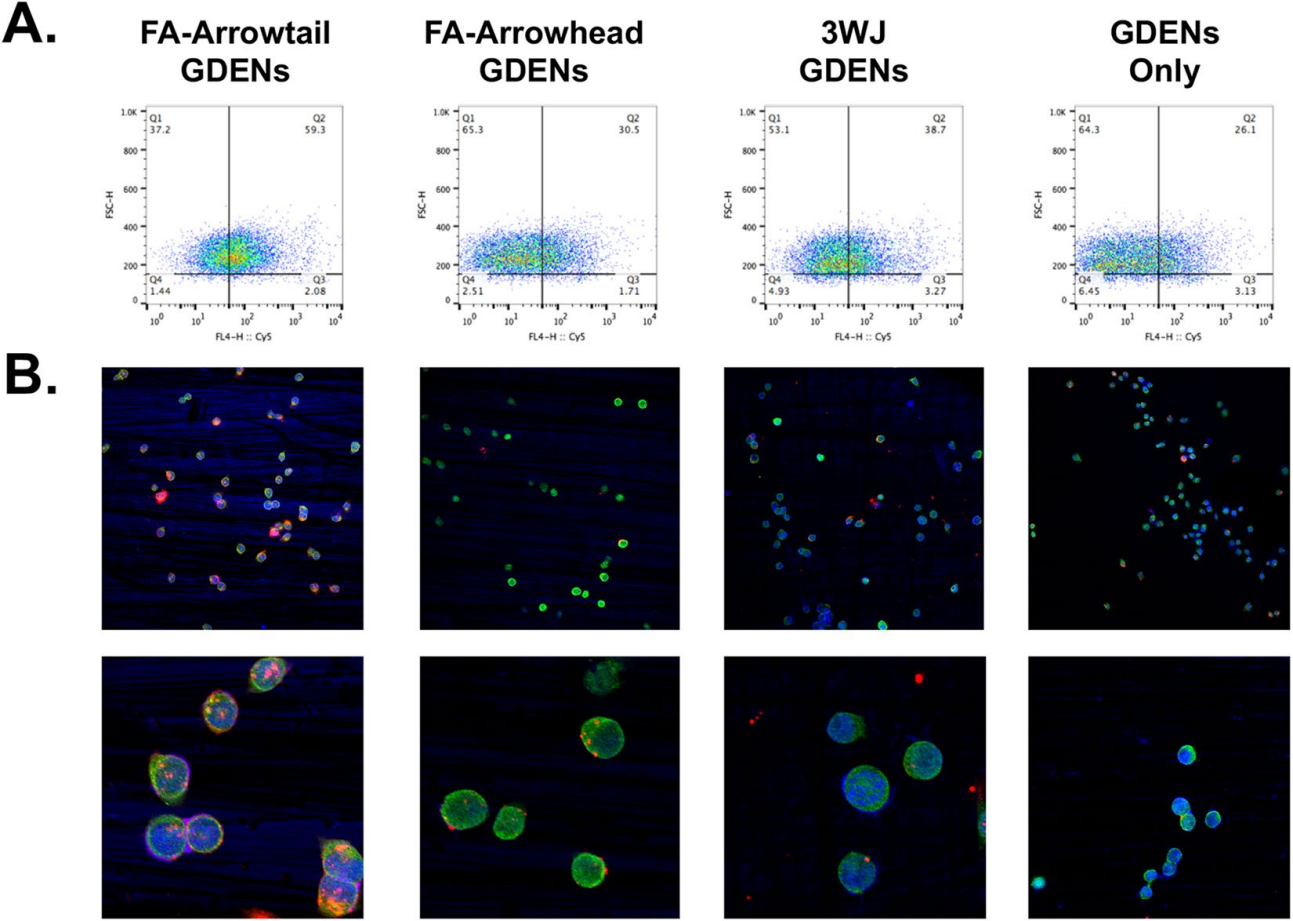
Cell binding and uptake of the arrowtail RNA ligand-displaying GDENs. (**A**) Flowcytometry and (**B**) confocal microscopy imaging compare binding and uptake of GDENs engineered by FA-pRNA-3WJ arrowtail and arrowhead to folate receptor overexpressed KB cells. Nucleus (blue), cytoplasm (green) and Alexa_647_ labeled RNA loaded in GDENs (red) signals.

To further distinguish the arrowtail and arrowhead configuration, a small molecule folate was chosen as a targeting ligand and conjugated on pRNA-3WJ for cell binding and uptake studies (Fig. 6). Alexa_647_ label RNA was preloaded in GDENs as an indicator then incubated with folate conjugated arrowtail, arrowhead (Fig. S5), and other control groups, then purified by ultracentrifugation. Cell binding assayed by flow cytometry indicated folate-conjugated arrowtail pRNA-3WJ enhanced the binding of GDENs to KB cells compared to arrowhead and other control groups (Fig. 6A) as well as those in folate receptor negative cell line **(**Fig. S6**)**. Confocal microscopy images further confirmed that the ligand displaying arrowtail pRNA-3WJ facilitated GDENs uptake by KB cells (Fig. 6B). Considering the result observed above together, we confirmed that the arrowhead and arrowtail pRNA-3WJ exhibit partially loading or ligand displaying function, respectively, on GDENs similar to that we previously reported^5^.

### Therapeutic RNA delivery and Tumor suppression by ligand-displaying GDENs

**Figure 7:**
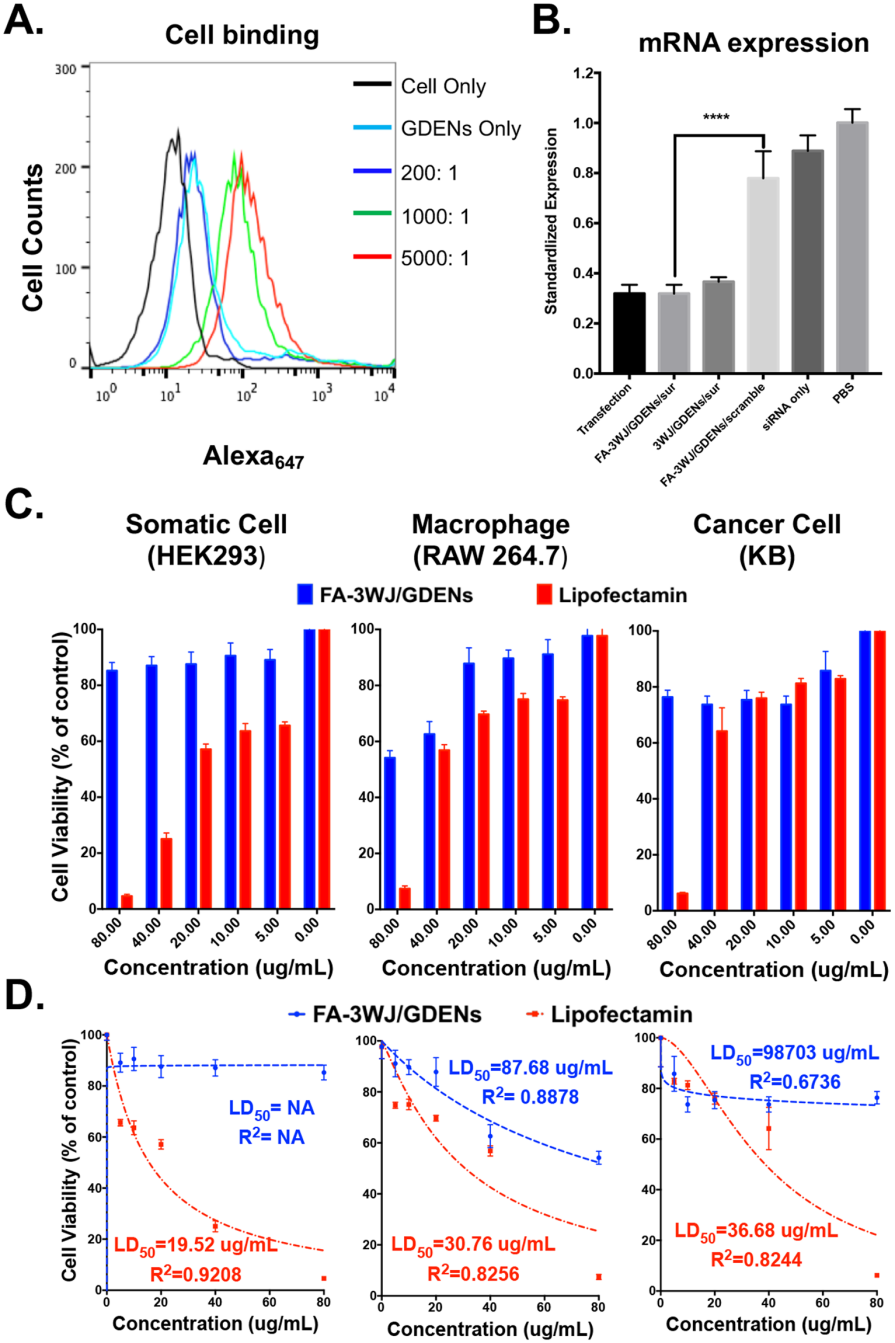
Cancer cell targeting and delivery of siRNA by ligand-displaying GDENs. (**A**) Cancer cell targeting capacity was evaluated by flowcytometry showed dose-dependent of FA-arrowtail RNA ratio verse equal amount of GDENs. (**B**) qRT-PCR evaluating *in vitro* delivery of survivin siRNA to KB cells by FA-arrowtail RNA ligand displayed GDENs. mRNA levels were standardized by PBS group as 1 and shown as mean ± S.D. fold changes of individual groups. Results are presented at n = 3 for each group for one-way ANOVA multiple comparisons, Tukey’s adjusted p of FA-3WJ/GDENs/sur compare to transfection, 3WJ/GDENs/sur and FA-3WJ/GDENs/scramble are >0.9999, 0.9174 and <0.0001(****). (**C**) Cytotoxicity of FA-3WJ/GDENs evaluated by MTT assay expressed as histogram and fit with nonlinear regression curve (**D**). Samples were incubated with cells in quadruplicate for 24 hours before adding MTT dye. Cell viability were standardized by the control group of “Cell Only” as 100%. Equivalent lipofectamin 2000 were used for comparison. N = 4, error bar indicates ± S.D.

FA displaying GDENs were then used to exam the targeting, delivery and gene silencing on KB cells and KB cells derived xenograft mice model. Increasing folate-arrowtail displaying ratio on GDENs from 200:1, 1000:1 and 5000:1 showed consecutive enhanced binding to KB cells (Fig. 7A). Delivery of survivin siRNA by FA-3WJ/GDENs/sisurvivin to KB cells was evaluated by examining the depleted expression of survivin at the mRNA level. Folate displaying GDENs exhibit similar delivery efficiency compared to transfected siRNA samples *in vitro* (Fig. 7B). The treatment group (FA-3WJ/GDENs/sisurvivin) exhibited significant gene knockdown effect compared to the control group treated with scramble RNA (FA-3WJ/GDENs/scramble) and the untreated group (PBS). Interestingly enough, we also observed gene knockdown effect on treatment group without ligand (3WJ/GDENs/sisurvivin), indicating that GDENs itself were taken in by cell and delivered the cargo into cell *in vitro*.

To evaluate the cytotoxicity of the FA-3WJ/GDENs as delivery vesicles, MTT assays were performed to study whether the nanoparticles will inhibit cell proliferation on somatic cell (HEK293), macrophage (Raw 264.7) and cancer cell (KB). To provide a standard, we used equivalent common transfection reagent (lipofectamine 2000) as reference. Cell proliferation remained more than 80% after 24 hours incubation with FA-3WJ/GDENs in 20 µg/mL, which is equal to the dose for *in vitro* delivery. Even for the highest dose (80 µg/mL), these nanoparticles showed significantly less cytotoxicity compared to the transfection reagent (Fig. 7C). By fitting the data with a non-linear regression curve, 50% lethal dose (LD_50_) were calculated (Fig. 7D). Macrophages were more sensitive to the nanoparticles, while considerably low cytotoxicity of FA-3WJ/GDENs was observed on both somatic and cancer cell. The overall results suggest the biocompatibility of FA-3WJ/GDENs as delivery vesicles.

**Figure 8:**
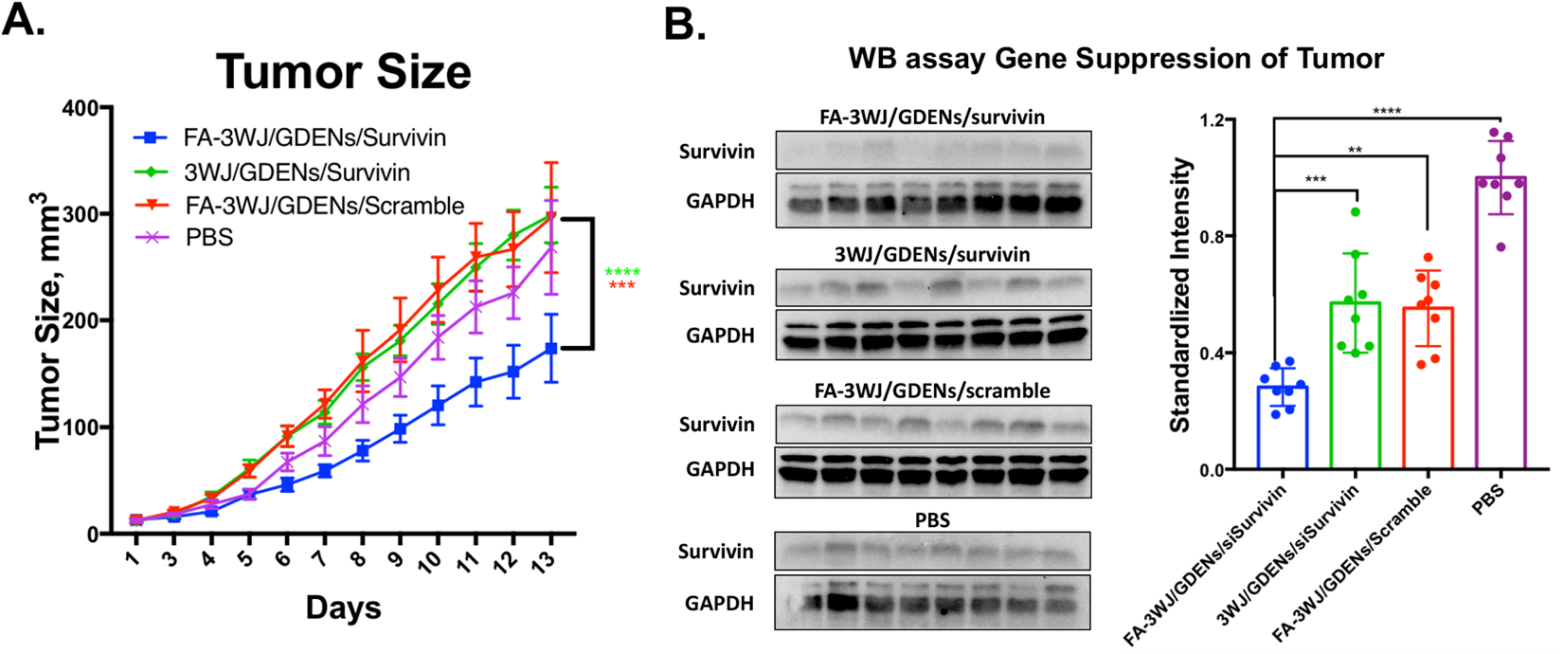
*In vivo* delivery of siRNA by ligand-displaying GDENs via IV injection. (**A**) Tumor size of nude mice with KB cell derived xenograft tumors by intravenously treatment every two days for two weeks. n = 8 and result is shown as mean ± S.E.M., analysis by multiple t-test. *p* = 0.0378, 0.0179, 0.0035, 0.0015, 0.0018, 0.0008 for day 8~13 post first dose given, respectively, for FA-3WJ/GDENs/siSurvivin v.s. FA-3WJ/GDENs/Scramble control. *p* = 0.0026, 0.0015, 0.0002, <0.0001, <0.0001, <0.0001 for day 8~13 post first dose given, respectively, for FA-3WJ/GDENs/siSurvivin v.s. 3WJ/GDENs/siSurvivin. (**B**) Western blot evaluating survivin protein level in tumor after intravenously treatment (All cropped blots were run under same condition and developed in same film, full blots are included in Supplementary Fig. S8). Gray value was quantified by Image J, intensity was adjusted by internal control GAPDH level and standardized by PBS group as 1. n = 8 and result is shown as mean ± S.D., analysis by one-way ANOVA, *p < 0.05, **p < 0.01, ***p < 0,001, ****p < 0.0001.

The *in vivo* delivery of survivin siRNA by folate-displaying GDENs was also evaluated in subcutaneous xenografts of KB cells. Upon tumor growth and maturation, FA-3WJ/GDENs/siSurvivin were delivered to the tumor through retro-orbital IV injection (1 dose every 2 days; total 6 doses). Delivery of RNA decorated GDENs showed suppressed tumor growth over a negative control (Fig. 8A). In addition, there were no significant body weight changes of the mice that were observed during treatment. This indicates that GDENs are biocompatible and no gross toxicity (Fig. S7). Upon the completion of the experiment, mice were sacrificed and tumor specimens were used to examine the protein level of *survivin* gene while using GAPDH as internal control. It was observed that siRNA loaded GDENs were able to significantly reduce *survivin* expressions within the tumor environment compared to both treatment scramble control (FA-3WJ/GDENs/scramble) and the treatment group without targeting ligand (3WJ/GDENs/sisurvivin) (Fig. 8B). The result from both *in vitro* and *in vivo* indicate that ligand-displaying GDENs by arrowtail RNA nanoparticle had potential as a delivery vector for therapeutic siRNA.

## Discussion

In this study, we demonstrated a strategy to isolate exosome-like nanovesicles from ginger as a siRNA delivery vesicle through intravenous administration. Ginger, as well as other edible plants, shows an economic advantage for the production of exosome-like vesicles on a large scale^13,14^. Here, we introduced equilibrium density gradient ultracentrifugation to increase the purity of GDENs while remaining ~300-fold lower cost/yield ratio to human cell derived EVs (Table S1). Compared to human cell derived EVs, plant exosome-like vesicle reduces not only the cost from cell culture supplies but also the time and labor of large scale cell culture. By using a normal juice blender, we can process up to 3 liters of ginger juice as a starting material in 1 hour, equivalent of 300 cell culture dishes (150 mm). Moreover, we haven’t yet reached the productivity limit as this was done within a research environment.

Cushion methods have been widely used in ultracentrifugation to avoid physical disruption caused by long ultracentrifugation time. Here, we provided evidences that adding a small volume of cushion for GDEN preparation can increase 2~3 fold in yield concerning both particle numbers and total protein concentration (Fig. 2b). When particles reach the bottom of the centrifuge tube to form a pellet, the cushion was buffered to protect the membrane and the individual free components (protein, lipid, nucleic acid, etc.) from ultra-high centrifuge force. They were not pelleted tightly due to an altered sedimentation coefficient. TEM imaging of GDENs by cushion method tends to have cleaner background, nicer spherical shape of GDENs, and less aggregation compared to traditional pelleting approach. Hence, we suggest cushion could be widely used in ultracentrifuge-based purification for EVs, exosome-like and other membrane vesicles.

Unlike EVs isolated from human cell cultures that naturally serve as intercellular carriers, GDENs may not carry such ligand and cargos that can be recognized by human cell. Recently, we reported that, by controlling the orientation of the arrowshape RNA nanoparticle, respective loading or surface display function on EVs can be achieved. In this study, we found similar properties when folate-conjugated arrowhead and arrowtail RNA particles were incubated with GDENs (Figs 5 and 7A).

IV injection is normally considered to have advantages in avoiding the first-pass effect of hepatic metabolism, producing the highest bioavailability. Here, we successfully administered the RNA/GDENs complex intravenously and show tumor inhibition in KB cell derived xenograft models. Unlike the siRNA delivery assay we performed *in vitro* (Fig. 7), the treatment with targeting ligand exhibited significant contribution to the therapeutic outcome when introducing IV given GDENs that encapsulated survivin siRNA (Fig. 8). One possible reason for these differences might be the cellular uptake speed. FA as a small molecule ligand can bring nanoparticles into the cell through the receptor mediated endocytosis pathway in 15~30 minutes^44^. Our flowcytometry and confocal imaging experiment were performed by 1-hour incubation, followed by a wash procedure. Aside from the significant enhance uptake by displaying FA ligand, we did observe GDENs bound without ligand compared to the cell only control group (Figs 6 and 7). We speculated that the GDENs were taken up non-specifically over long incubation time during *in vitro* assay. However, under an *in vivo* environment, the local concentration of nanoparticles in tumor is expected to be much lower and dynamic due to clearance effect. Thus, GDENs without ligand were much less likely to be taken up by cancer cells *in vivo*, which leaded to lack of therapeutic outcome and significantly less gene knocked down compared to FA-arrowtail decorated GDENs.

Interestingly, tumors in the two negative control groups tend to grow faster compared to PBS control; however, statistical significance was not observed. One possible reason might be the overall slower tumor growth rate with folate deficient diet was rescued by certain growth factors contained in GDENs^45,46^. Follow-up studies on the effect of blank GDENs may be valuable since ginger has been widely used in food consumption as well as herb and nutrition supplement, and several studies also reported that ginger derived exosome-like nanoparticles had treatment effect in different disease models by oral administration^17,47^. Moreover, there is no significant weight loss of the mice during 6 repeat dosing (Fig. S7), which indicate GDENs, as well as other plant exosome-like vesicles, could be biocompatible as a systematic delivery system. Nevertheless, further research, especially immunogenicity and toxicity of GDENs, needs to be done in order to address the concern of biocompatibility since current knowledge regarding the side effects of plant exosome-like vesicles are very limited when systematically introduced.

## Methods

### GDENs purification from Ginger roots

Ginger roots were purchased in a local fresh market and washed thoroughly by 1 × PBS, then blended under maximum power in cold room for 10 min (1 min on, 1 min pause). Blended juice was passed through a gauze bucket to exclude rough residues then centrifuge under 10,000 g at 4 °C by Fiberlite^TM^ F12–6 × 500 LEX Fixed Angle Rotor for 1 hour, keeping only the supernatant, and repeated 2~3 times. 100,000 g ultracentrifugation was performed using a SW28 rotor (*Beckman Coulter*) for 80 min at 4 °C to concentrate GDENs. 200 ul Optiprep (60% iodixanol, *Sigma*) was added to the bottom of each tube to serve as iso-osmotic cushion. The supernatant was carefully removed from the top and around 2 mL of the fraction close to the interface and cushion were collected. Every 12 mL GDEN solution was further concentrated to 2 mL fraction by repeated 100,000 g ultracentrifugation. The GDEN solution then mixed with 60% iodixanol in different ratio to form 10% and 40% iodixanol solution. 2.5 mL of each solution were added in a SW55 ultracentrifuge tube to form two separate fractions. An equilibrium density gradient was formed by 24 hours 40,000 rpm ultracentrifugation at 4 °C. 24~25 fractions were then collected for further analysis from the bottom of each tubes by fraction collector we developed in our lab.

### Density measurement

Density of collected fractions was measured following standard protocol by manufacture. In brief, fractions sample were 1:5000 diluted with ddH_2_O and measure OD_244_ by Nanodrop 2000, then converted by the standard curve as shown in Figs 1B and S1.

### Nanoparticle Tracking Analysis (NTA), total protein concentration measurement and EM imaging

NTA was performed using the Malvern NanoSight NS300 system on GDENs fractions and dilute 400~2000 folds with 1 × PBS to obtain optimal signal count per frame according manufacturer instruction (30~50 reads/frame). Three 60-second videos were recorded and analyzed by NTA software.

Total protein concentration was measured by BCA assay following manufacturer’s instruction. In brief, 10 µL of protein sample were mixed with 200 µL BCA reagent alongside with Albumin standard (0.025~2 µg/µL) then incubate in 96-well plate at 37 °C for 25 min. OD_562_ were measured by plate reader (BioTek). Linear standard curve was plotted to convert OD_562_ to protein concentration.

EM imaging followed the typical negative staining methods reported for EVs^48^. In short, Copper grids (400 mesh, TED PELLA) were immersed into GDENs for 5 min and then stain with 1% uranyl acetate for 30 s. Imaging was taken by FEI Tecnai G2 Spirit TEM at the Campus Microscopy & Imaging Facility (CMIF) in Ohio State University.

### RNA nanoparticle design, synthesis, and self-assembly

RNA strands for assembled RNA nanoparticles were designed and synthesized following our previously published methods^5,49^. RNA nanoparticles were then self-assembled in a one-pot manner by mixing individual RNA strands at equimolar concentrations (25 μM) in 1× PBS buffer (137 mM NaCl, 10 mM Phosphate, 2.7 mM KCl, pH 7.4) and heated to 95 °C for 10 minute, followed by slowly (1 hour) cooling down to 4 °C.

### RNA loading/decorating GDENs, GDENs membrane labeling, loading efficiency evaluation and Serum degradation assay

GDENs (0.05 pmole) and Alexa_647_ fluorescent conjugated RNA (0.5 nmole) were mixed in 100 μl of 1 × PBS with 2.5 μl of ExoFect Exosome transfection (System Biosciences) following the manufacturer’s procedures. Unloaded RNA from the supernatant was collected and Alexa_647_ intensity on supernatant was measured to evaluate loading efficiency compared with control groups. Arrowhead or arrowtail RNA nanoparticles were incubated with GDENs at 37 °C for 1 hour, chilled on ice until usage. RNA to GDENs ratios were calculated based on molar concentration using a 5000:1 ratio for siRNA delivery assay for both *in vitro* and *in vivo*. For labeling the GDENs membrane, we mixed 10X concentration of *CellMask*^*TM*^ *Orange* (*Invitrogen*) as manufacture defined with 0.5 nM GDENs to 1X concentration and incubate at 37 °C for 30 min in dark. All engineered GDENs described ahead were purified by cushion ultracentrifugation methods mentioned above. Serum digestion was performed by mixing the purified engineered GDENs with Fetal Bovine Serum (*Sigma-Aldrich*) to 67%, then incubate in 37 °C for 5 min, 2 hours and 4 hours, then run in 2% Synergel for electrophoresis in TAE (40 mM tris-acetate, 1 nM EDTA) buffer to test the degradation pattern. Gel was imaged by the Typhoon (GE Healthcare).

### Cell Culture

KB cells (ATCC CCL-17) were pre-cultured in folic acid depleted RPMI 1640 Medium with 10% Fetal Bovine Serum (Sigma) for three days before cell uptake, *in vitro* delivery assay and tumor xenograft.

### Flow Cytometry and Confocal Microscopy Imaging

For FACS, 50 nM Alexa647 labeled-RNA loaded GDENs were incubate with 1 × 10^5^ KB cells in 100 μl volume at 37 °C for 1 hr. After washing twice with 1× PBS, cell fluorescence analysis was done by BD FA CSCalibur^TM^ flow cytometry system at the Analytical Cytometry Shared Resource (ACSR) in the OSU comprehensive Cancer Center (OSUCCC).

For confocal microscopy imaging, KB cells were seeded on glass coverslips and cultured at 37 °C overnight. 50 nM RNA loaded GDENs were incubated with cells at 37 °C for 1 hr. After washing twice with 1× PBS, cells were fixed with 4% formaldehyde, then staining with *Alexa Fluor® 488 phalloidin* (Life Technologies Corporation, Carlsbad, CA) for cytoskeleton and mount with *Fluoroshield Mounting Medium With DAPI* (abcam) for cell nucleus. Confocal images were taken by Olympus FV3000 confocal microscope (Olympus Corporation, Tokyo, Japan) at the CMIF in Ohio State University.

### Assay for mRNA expression by qRT-PCR

For *in vitro* delivery assay, 5 × 10^4^ of KB cells were plated in 24-well plates 1 day ahead treatment and maintained at 37 °C in folate free medium. Cells were washed twice with 1 × PBS and then treated with 50 nM of either FA-3WJ/GDENs/3WJ-siSurvivin or negative controls in serum free medium for 4 hours then switched to full medium and continued to culture for 72 hr. Cells were then washed with 1 × PBS three times and total RNA was extracted using TRIzol RNA extraction reagent (Thermo Fisher Scientific) following the manufacturer’s instruction. Using SuperScript III First-Strand Synthesis (Invitrogen), 1 μg total RNA was used in reverse transcription producing. TaqMan real-time PCR was perform following manufacturer’s procedures; samples were run in triplicate. Primers/probes set for human BIRC5 and 18S were purchased from Life Technology. PCR was performed on Step-One Plus real-time PCR system (Applied Biosystem). The raw data obtained was analyzed using the comparative CT Method (ΔΔCT Method).

### Assay for cytotoxicity evaluation by MTT

To evaluate cytotoxicity of FA-3WJ/GDENs, 1 × 10^4^ of HEK293 cells, Raw. 264.7 macrophage and KB cells were seeded in individual 96-well plates 1 day ahead treatment and maintained at 37 °C in 100 µL full medium. FA-3WJ/GDENs and lipofectamine were diluted and added to cells in quadruplicate to a final concentration 80 µg/mL then followed 2-fold series dilution to 40, 20, 10 and 5 µg/mL groups. After 24 hours incubation, 15 µL MTT dye (Promega) were add to individual well then incubate in dark for 4 hours. 100 µL Solubilization solution/Stop Mix (Promega) were add to individual well for dissolving the crystal in dark. Once crystal fully dissolved, OD_570_ were measured by plate reader (BioTek) following manufacture procedures. Untreated groups were used for standardization as 1 and data were process and fit with non-linear regression in Prism 7.

### *In vivo* tumor regression assay in xenograft mice model

To generate KB cell xenograft mice model, female athymic nude Nu/Nu (6–8 weeks old) mice (Taconic) were used. Subcutaneous xenografts were creating by injecting 2 × 10^6^ KB cells in 100 μl of 1 × PBS into each mouse. Once tumor volumes reached of ~100 mm^3^, the mice were anesthetized using isoflurane gas (3% in oxygen at a flow rate of 0.6 l·min^−1^) and injected intravenously through retro-orbital injection with 6 repeat doses of 0.1 pmole GDENs/0.5 nmole RNA per mice every two day. The protocol for this animal experiment was approved by the Institutional Animal Care and Use Committee (IACUC) of the Ohio State University. All animal procedures were housed and performed in accordance with the Subcommittee on Research Animal Care of The Ohio State University guidelines approved by the Institutional Review Board.

### Assay for protein expression by Western Blotting

Tumor specimens were sliced to small pieces and homogenized in 200 µL RIPA buffer (with protease inhibitor cocktail), insoluble materials were removed by centrifugation at 12,000 g for 10 min at 4 °C. After measuring total protein concentration by BCA method, 15 µg of individual sample of 4 groups were loaded into 4 12% SDS-PAGE gel with Spectra™ Multicolor Broad Range Protein Ladder (Thermo Fisher) and then run in Mini-PROTEAN® Tetra Cell Systems (Bio-rad). Proteins were then transferred to Immun-Blot PVDF Membrane(Bio-rad) by Trans-Blot® Turbo™ Transfer System (Bio-rad) in 25 V, 1.3 mA (constant current) for 5 min. The membrane was blocked in 5% fat-free milk in room temperature for 2 hours and then incubated with anti-survivin **(**Rb mAb, 1:5000 diluted in 2% milk, Abcem**)** and anti-GAPDH (Rb mAb, 1:1000 diluted in 2% milk, ProSci) in 4 °C for overnight. TBST was used to wash the membrane for 10 min × 3 times and then the membrane was incubated with secondary antibody (Goat pAb to Rb IgG, 1:20000 diluted in 2% milk, Abcem) at room temperature for 40 min then the TBST wash step was repeated. 4 membranes were then incubated together with ECL substrate (Bio-rad) for 3 mins. Membranes were then exposed to Amersham Hyperfilm^TM^ (GE Healthcare) together and process by Series 2000A Processor film developer (TiBA). Film were scanned (Epson V550) and quantify by ImageJ 2.0.

### Statistics

Each experiment was performed at for least three biological repeats with triplication for each sample tested. The results were presented as mean ± standard deviation, unless otherwise indicated. Statistical mean differences were evaluated using unpaired t-test or ANOVA (p value adjusted for multiple comparisons by Tukey’s procedure with GraphPad software, and p < 0.05 was considered statistically significant).

## Electronic supplementary material

Supplementary Information

## Data Availability

The datasets generated during and/or analyzed during the current study are available from the corresponding author on reasonable request.
