# Supplementary material for: Arrowtail RNA for Ligand Display on Ginger Exosome-like Nanovesicles to Systemic Deliver siRNA for Cancer Suppression
Source: Sci Rep. 2018 Oct 2;8:14644. doi: 10.1038/s41598-018-32953-7 (PMC6168523; doi:10.1038/s41598-018-32953-7)
Supplement: Supplementary file 1 — Supplementary Information [file 41598_2018_32953_MOESM1_ESM.docx]

***Supplementary:***

**Arrowtail RNA for Ligand Display on Ginger Exosome-like Nanovesicles to Systemic Deliver siRNA for Cancer Suppression**

Zhefeng Li^1^, Hongzhi Wang^1^, Hongran Yin^1^, Chad Bennett^2^, Huang-ge Zhang^3^, Peixuan Guo^1*^

^1^*Center for RNA Nanobiotechnology and Nanomedicine; College of Pharmacy, Division of Pharmaceutics and Pharmaceutical Chemistry; College of Medicine; Dorothy M. Davis Heart and Lung Research Institute; NCI Comprehensive Cancer Center; The Ohio State University, Columbus, OH 43210, USA*

^2^Medicinal Chemistry Shared Resource, Comprehensive Cancer Center; The Ohio State University, Columbus, OH 43210, USA

*^3^James Brown Cancer Center, Department of Microbiology & Immunology, University of Louisville, Louisville, KY, USA*

*Correspondence should be addressed to:

Peixuan Guo, PhD ([guo.1091@osu.edu](file:///C:\Users\Farzin%20Haque\Desktop\Manuscripts%20In%20progress\Hui%20Li%20-%20Tetrahedron\guo.1091@osu.edu))

Sylvan G. Frank Endowed Chair in Pharmaceutics and Drug Delivery

The Ohio State University

912 Biomedical Research Tower (BRT), 460 W 12th Ave., Columbus, OH 43210, USA

**Figure S1**.

**Relative to Fig.1B**

**Figure S2**. Size distribution of fractions measured by NTA.

**Relative to Figure 1B**

**Figure S3.**

**DLS measurement of GDENs**


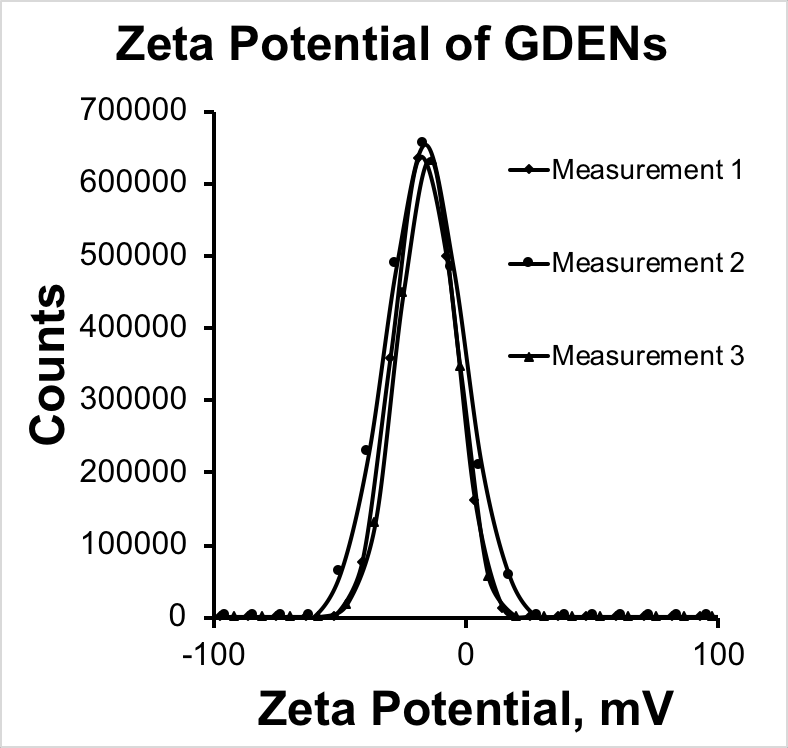


**Figure S4.**

**Relative to Figure 3C**

**Figure S5.**

**Figure S6.** Flowcytometry show negative cell binding in folate receptor (-) HEK293 cell.

**Relative to Figure 6A**

**Figure S7.** Weight monitoring of mice during 2 weeks treatment.

**Figure S8**

**Relative to Fig. 8B**

**Table S1**. Cost and yield calculation.
